# Viscoelastometry for detecting oral anticoagulants

**DOI:** 10.1186/s12959-021-00267-w

**Published:** 2021-03-16

**Authors:** Philipp Groene, Daniela Wagner, Tobias Kammerer, Lars Kellert, Andreas Giebl, Steffen Massberg, Simon Thomas Schäfer

**Affiliations:** 1 Department of Anaesthesiology, University Hospital Munich, LMU Munich, Marchioninistraße 15, 81377 Munich, Germany; 2 Department of Anaesthesiology and Intensive Care Medicine, University Hospital of Cologne, Cologne, Germany; 3 Department of Neurology, University Hospital Munich, LMU Munich, Munich, Germany; 4 Department of Transfusion Medicine and Hemostaseology, University Hospital Augsburg, Augsburg, Germany; 5 Department of Internal Medicine I – Cardiology, University Hospital Munich, LMU Munich, Munich, Germany

**Keywords:** Anticoagulants, Coagulation tests, Dabigatran, Factor Xa (FXa) inhibitors, Heparin, Viscoelastometry

## Abstract

**Background:**

Determination of anticoagulant therapy is of pronounced interest in emergency situations. However, routine tests do not provide sufficient insight. This study was performed to investigate the impact of anticoagulants on the results of viscoelastometric assays using the ClotPro device.

**Methods:**

This prospective, observational study was conducted in patients receiving dabigatran, factor Xa (FXa)-inhibitors, phenprocoumon, low molecular weight heparin (LMWH) or unfractionated heparin (UFH) (local ethics committee approval number: 17–525-4). Healthy volunteers served as controls. Viscoelastometric assays were performed, including the extrinsic test (EX-test), intrinsic test (IN-test) Russel’s viper venom test (RVV-test), ecarin test (ECA-test), and the tissue plasminogen activator test (TPA-test).

**Results:**

70 patients and 10 healthy volunteers were recruited. Clotting time in the EX-test (CT_EX-test_) was significantly prolonged versus controls by dabigatran, FXa inhibitors and phenprocoumon. CT_IN-test_ was prolonged by dabigatran, FXa inhibitors and UFH. Dabigatran, FXa inhibitors and UFH significantly prolonged CT_RVV-test_ in comparison with controls (median 200, 207 and 289 vs 63 s, respectively; all *p* < 0.0005). Only dabigatran elicited a significant increase in CT_ECA-test_ compared to controls (median 307 vs 73 s; *p* < 0.0001). CT_ECA-test_ correlated strongly with dabigatran plasma concentration (measured by anti-IIa activity; r = 0.9970; p < 0.0001) and provided 100% sensitivity and 100% specificity for detecting dabigatran. Plasma concentrations (anti-XA activity) of FXa inhibitors correlated with CT_RVV-test_ (r = 0.7998; *p* < 0.0001), and CT_RVV-test_ provided 83% sensitivity and 64% specificity for detecting FXa inhibitors.

**Conclusions:**

In emergency situations, ClotPro viscoelastometric assessment of whole-blood samples may help towards determining the presence and type of anticoagulant class that a patient is taking.

**Trial registration:**

German clinical trials database ID: DRKS00015302.

**Supplementary Information:**

The online version contains supplementary material available at 10.1186/s12959-021-00267-w.

## Background

Patients with pre-existing cardiovascular disease are at increased risk of thromboembolic events. Considering the trend towards increased longevity among human populations across the world, the use of anticoagulation therapy has become widespread [1, 2]. A range of effective treatments are available, but patients receiving anticoagulation therapy can be at risk of excessive bleeding, e.g. in trauma or emergency surgery [3–5]. These situations may require immediate treatment of anticoagulant effects, whereas interventions differ between anticoagulants [6, 7]. Depending on urgency, vitamin K antagonists (VKAs, e.g. warfarin) may be reversed using vitamin K or prothrombin complex concentrates (PCCs) [8, 9]. Furthermore, specific reversal agents have been developed for direct oral anticoagulants (DOACs), including idarucizumab for direct thrombin inhibitors and andexanet alfa for emergency bleeding situations in patients on direct FXa- antagonists [9, 10].

Thus, in an unknown patient with emergency bleeding, it may be necessary to determine the presence and type of anticoagulant therapy by performing coagulation tests. Fast, reliable detection and differentiation are essential in patients with severe bleeding, to enable administration of the most effective reversal therapy (e.g. antagonization by specific antidotes such as andexanet alfa or idarucizumab). The results of standard laboratory tests (e.g. international normalized ratio [INR], activated partial thromboplastin time [aPTT]), as well as standard viscoelastic tests (e.g. EXTEM clotting time (CT), TEG R-time), are affected by anticoagulant drugs [11–14]. However, these routine tests do not readily provide sufficient insight to determine exactly which drug a patient is taking. Anti-factor Xa and anti-factor IIa tests may help determine presence of a factor Xa inhibitor or a direct thrombin inhibitor but these laboratory assessments are affected by heparin and they have prolonged turnaround times. There is a need for specific diagnostic tools suitable for point-of-care use in emergency situations [15, 16].

A newly developed viscoelastometric device (ClotPro, enicor GmbH, Munich, Germany) using different methodology from ROTEM or TEG, provides rapid bedside evaluation of whole-blood coagulation [17, 18]. Multiple assays can be performed concurrently as the device has six test channels [17]. Some assays are comparable with commonly used ROTEM/TEG assays (e.g. extrinsic test [EX-test], functional fibrinogen test [FIB-test], intrinsic test [IN-test]). Additional assays have been developed specifically for the detection and differentiation of anticoagulants: the Russel’s viper venom test (RVV-test) and the ecarin test (ECA-test). We performed a trial to assess the effects of anticoagulant drugs, including DOACs, on viscoelastometric test results.

## Methods

This in vivo, prospective, observational, single-center study was conducted between November 2018 and October 2019. It was registered at the German clinical trials database (ID: DRKS00015302), and the protocol was approved by the Ludwig-Maximilians-University ethics committee (No 17–525-4). The study was performed in accordance with the Declaration of Helsinki, with written informed consent being obtained from all participants before they undertook any of the study procedures.

Adults who had been treated with any one of the following drugs for at least 7 days were eligible to participate: dabigatran, rivaroxaban, apixaban, edoxaban, phenprocoumon, low molecular weight heparin (LMWH) or unfractionated heparin (UFH). In each of these seven treatment groups, concurrent treatment with platelet inhibitors (e.g. acetylsalicylic acid or clopidogrel) was permissible. A control group of healthy volunteers was recruited, bringing the total number of study groups to eight. Exclusion criteria were as follows: age < 18 years, treatment with more than one anticoagulant, coagulation disorders (e.g. von Willebrand’s disease), and myelodysplastic syndrome.

Each study participant provided a single blood sample for analysis. To reflect the clinical circumstances of patients in whom emergency bleeding may occur at any time, no measures were taken to control the time at which samples were taken.

All samples were tested using a ClotPro device (enicor GmbH, Munich, Germany) within 2 h after blood withdrawal, under standardized conditions (37 °C, maximum test run time 4.500 s). Five assays were performed on each sample (EX-test, IN-test, ECA-test, RVV-test and tissue plasminogen activator test [TPA-test]), and the following variables were documented: CT, clot formation time (CFT), clot amplitude at 5 and 10 min after CT (A5, A10), maximum clot firmness (MCF), maximum lysis (ML), as well as lysis time (LT; time from CT until 50% lysis) and lysis onset time (LOT; time from CT until 15% lysis) for TPA-test. Standard laboratory tests were performed on each sample: INR (Thromborel S; Siemens Healthcare GmbH, Erlangen, Germany), thrombin time (TT) (Berichrom Thrombinreagenz; Siemens Healthcare GmbH, Erlangen, Germany), aPTT (Actin FSL; Siemens Healthcare GmbH, Erlangen, Germany) and full blood count. Anti-activated factor X (anti-Xa) and anti-activated factor II (anti-IIa) tests were also performed, using Biophen Heparin LRT 7.5 (Hyphen Biomed, Neuville-sur-Oise, France).

Quality controls were performed according to institutional and manufacturers standards.

### Statistical considerations

10 individuals per study group were recruited (*N* = 80). This sample size was calculated from one-way analysis of variance of the magnitude of expected differences in CT between the oral anticoagulants included in the study and healthy controls. A large difference between the anticoagulants and healthy controls was assumed (effect size f = 0.4; alpha error 0.05; power 0.8). The software used was G*Power 3.1 (provided by the University of Düsseldorf). This procedure was done due to preliminary work on in-vitro dose-effect curves with DOACs and the new tests.

Statistical analyses of the results were performed using Graph Pad Prism 8 (La Jolla, USA) and SPSS Version 25 (IBM, Armonk, USA). All data are presented as median with interquartile range (IQR; 25th–75th percentile) unless indicated otherwise. For statistical comparison of study groups, results from the three activated factor X (FXa) inhibitors were pooled. Differences between groups were analyzed using the Kruskal-Wallis-test and Dunn’s multiple comparison test. The significance level (alpha) was adjusted for multiple testing (*p* = 0·05/n). Associations between variables were assessed using Spearman’s correlation coefficient.

To investigate the detection of an anticoagulant drug, all samples from patients who had taken the drug were compared against samples from controls. Receiver operating characteristic (ROC) analyses were performed to evaluate the sensitivity and specificity of test parameters for detecting anticoagulant drugs. Optimal cut-off values were determined by Youden’s Index.

## Results

A total of 80 individuals were recruited for the study, and their characteristics are shown in Table 1. The last intake of anticoagulant medication was at a median of 3.75 h (range, 1.00–12.00) before taking the blood sample in patients treated with dabigatran, 6.00 h (range, 0.50–17.50) among those receiving FXa inhibitors, and 3.25 h (range 0.50–13.50) in patients on LMWH.

**Table 1:** Demographics and other characteristics of the study participants. Data are presented as median (interquartile range), unless indicated otherwise

	Controls (***n*** = 10)	Rivaroxaban (n = 10)	Apixaban (n = 10)	Edoxaban (n = 10)	Dabigatran (n = 10)	Phenprocoumon (n = 10)	LMWH (n = 10)	UFH (n = 10)
**Female/male gender, n (%)**	6/4 (60/40)	5/5 (50/50)	8/2 (80/20)	9/1 (90/10)	8/2 (80/20)	6/4 (60/40)	6/4 (60/40)	3/7 (30/70)
**Age (years)**	60 (58–64)	71 (54–81)	82 (79–87)	77 (73–83)	72 (58–79)	82 (45–86)	70 (42–79)	74 (63–82)
**BMI (kg/m**^**2**^**)**	27.1 (25.6–30.7)	23.7 (22.0–31.7)	23.9 (23.2–26.0)	27.0 (24.0–29.2)	26.3 (23.6–29.5)	26.6 (24.6–30.2)	24.3 (20.3–28.1)	26.6 (22.0–27.9)
**ASA status, n (%)**								
1	2 (20)	0	0	0	0	0	0	0
2	8 (80)	2 (20)	0	0	3 (30)	0	1 (10)	0
**3**	0	8 (80)	10 (100)	10 (100)	7 (70)	10 (100)	9 (90)	9 (90)
**4**	0	0	0	0	0	0	0	1 (10)
**Diabetes, n (%)**								
**Yes**	0	2 (20)	2 (20)	2 (20)	2 (20)	4 (40)	1 (10)	3 (30)
**No**	10 (100)	8 (80)	8 (80)	8 (80)	8 (80)	6 (60)	9 (90)	6 (70)
**Creatinine (mg/dL)**	0.9 (0.78–0.95)	1.0 (0.89–1.49)	1.2 (1.2–1.4)	1.2 1.1–1.4)	1.1 (0.9–1.3)	1.5 (1.1–2.6)	1.1 (0.88–1.4)	1.0 (0.6–2.5)
**GFR (ml/min)**	96 (93–106)	79 (65–99)	56 (48–82)	71 (52–81)	74 (64–104)	61 (35–84)	92 (70–112)	73 (23–86)
**Platelets (10**^**9**^**/L)**	262 (210–289)	214 (141–265)	247 (159–294)	220 (180–271)	229 (184–293)	239 (170–266)	282 (211–330)	227 (156–350)
**Hb (mg/dL)**	14.3 (13.7–15.2)	12.5 (10.0–14.6)	11.8 (9.9–13.6)	13.1 (11.9–14.1)	13.6 (11.4–15.1)	12.2 (10.7–15.3)	11.0 (9.4–12.6)	8.3 (7.4–9.1)
**Hematocrit**	0.41 (0.39–0.43)	0.38 (0.31–0.43)	0.36 (0.3–0.41)	0.38 (0.37–0.41)	0.40 (0.34–0.44)	0.36 (0.32–0.45)	0.31 (0.28–0.35)	0.26 (0.22–0.28)
**Fibrinogen (mg/dL)**	367 (325–411)	413 (359–447)	530 (366–594)	481 (310–570)	365 (298–425)	420 (390–500)	429 (341–597)	414 (319–509)

*ASA* American society of anesthesiologist status; *BMI* body mass index; *GFR* glomerular filtration rate; *Hb* hemoglobin; *LMWH* low molecular weight heparin; *UFH* unfractionated heparin

**Fig. 1:**
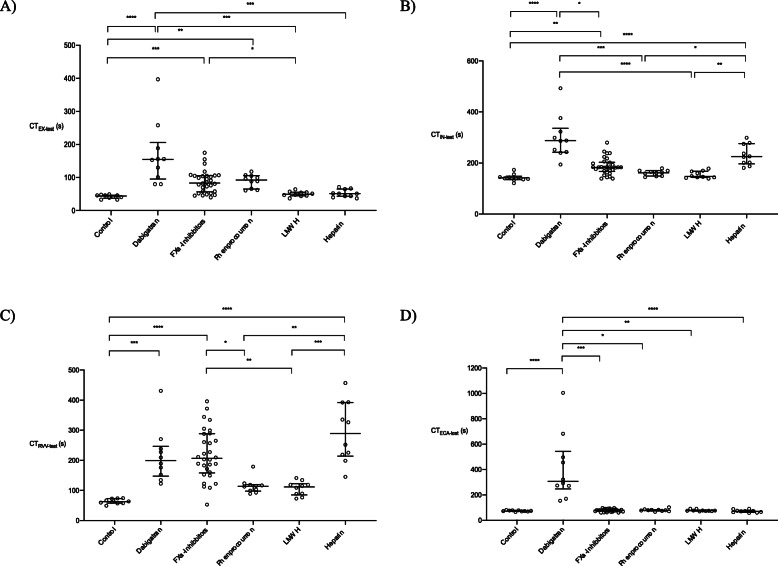
Viscoelastometric CT results (scatter dot plots) from the control group and the anticoagulant groups. Data are shown as median and interquartile range. **a** EX-test, **b** IN-test, **c** RVV-test and **d** ECA-test. * p < 0.05, ** *p* < 0.01, *** *p* < 0.001, **** *p* < 0.0001. CT, clotting time; LMWH, low molecular weight heparin

Median CT_EX-test_ was 44 s (IQR 38–47 s) in the control group (see Fig. 1a, Supplemental Tables 1A and B). Significant prolongation of CT_EX-test_ was observed with several of the anticoagulants: dabigatran, FXa inhibitors except for apixaban, and phenprocoumon. Dabigatran produced the largest increase in CT_EX-test_: median value 155 s (IQR 96–206 s); *p* < 0.0001 versus controls. CT_EX-test_ was not significantly prolonged versus controls by UFH or LMWH. None of the anticoagulant drugs produced statistically significant changes in A5_EX-test_ or MCF_EX-test_ compared to the control group (Supplemental Table 1A). In the ROC analysis, the optimal CT_EX-test_ cut-off for FXa-inhibitor detection was determined as 69 s. This cut-off was associated with an AUC of 0.649 (standard error [SE], 0.062), sensitivity of 70%, and specificity of 64%.

In the control group, the median CT_IN-test_ was 142 s (IQR 135–148 s; Fig. 1b, Supplemental Tables 1A and B). Statistically significant prolongation of CT_IN-test_ was seen with dabigatran and FXa inhibitors except for apixaban. Unlike with CT_EX-test_, UFH also produced significant prolongation of CT_IN-test_. The optimal CT_IN-test_ cut-off for UFH was determined as 186 s. This cut-off was associated with an area under the curve (AUC) of 0.768 (SE 0.056) and a sensitivity of 90% and a specificity of 67%.

CT_RVV-test_ in controls was 63 s (IQR 58–73 s; Fig. 1c, Supplemental Tables 1A and B). Significantly increased values for this parameter were observed with UFH, FXa inhibitors and dabigatran. The largest increase in CT_RVV-test_ was observed with UFH (median 289 [214–392] seconds; *p* < 0.0001 versus controls). Correlations were observed between CT_RVV-test_, and plasma FXa-inhibitor concentrations measured by anti-Xa activity (rivaroxaban: r = 0.5636, *p* = 0.0963; apixaban: r = 0.5714, *p* = 0.2), and statistical significance was reached with edoxaban (r = 0.9394; *p* = 0.0002 and pooled FXa inhibitors: r = 0.7998; p < 0.0001). CT_RVV-test_ also showed statistically significant correlation with anti-Xa activity of samples from patients in the LMWH group (r = 0.6687; *p* = 0.0395). In the ROC analysis, the optimal CT_RVV-test_ cut-off for FXa-inhibitor detection was determined as 147 s. This cut-off was associated with an AUC of 0.721 (standard error [SE], 0.058), sensitivity of 83%, and specificity of 64%. T Detection of apixaban (apixaban vs. controls) was improved using RVV-test (90% sensitivity and 100% specificity) compared to EX-test (80% sensitivity and 80% specificity).

The optimal CT_RVV-test_ cut-off for UFH was determined as 194 s. This cut-off was associated with an AUC of 0.808 (SE 0.064) and a sensitivity of 90% and a specificity of 63%.

Dabigatran was the only anticoagulant to produce a significant change versus control in CT_ECA-test_ (median 307 [246–543] vs 73 [70–78] seconds, respectively; *p* < 0.0001 Fig. 1d, Supplemental Tables 1A and B). Dabigatran plasma concentrations (measured by anti-IIa activity) were strongly correlated with CT_ECA-test_ (r = 0.9970; p < 0.0001). The optimal cut-off value of CT_ECA-test_ for detecting dabigatran was calculated as 128 s. This yielded an AUC for the ROC curve of 1.00 (SE, 0.00), with 100% sensitivity and 100% specificity.

**Fig. 2:**
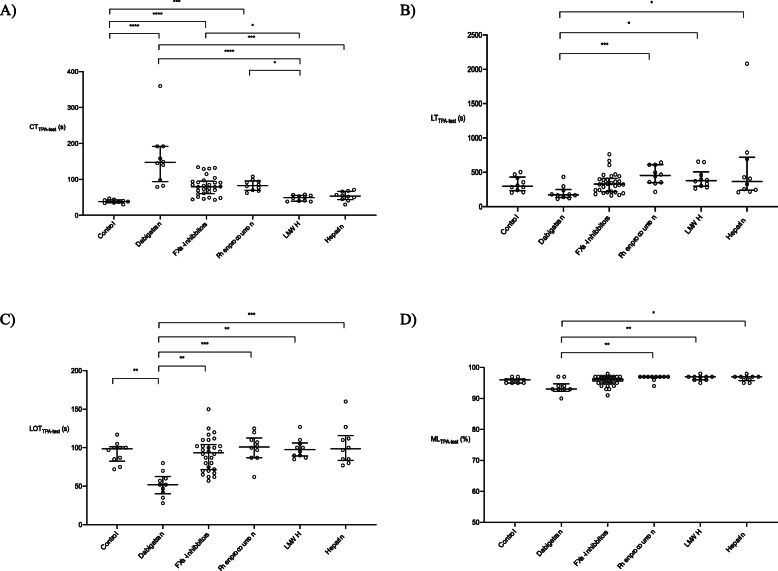
Viscoelastometric TPA-test results (scatter dot plots) from the control group and the anticoagulant groups. Data are shown as median and interquartile range. **a** CT, **b** LT, **c** LOT and **d** ML. * *p* < 0.05, ** *p* < 0.01, *** *p* < 0.001, **** *p* < 0.0001. CT, clotting time; LT, lysis time; LOT, lysis onset time; ML, maximum lysis; LMWH, low molecular weight heparin

In all eight study groups, median values for CT_TPA-test_ were numerically comparable to those for CT_EX-test_. Accordingly, as with CT_EX-test_, CT_TPA-test_ was significantly prolonged versus controls by dabigatran, FXa inhibitors and phenprocoumon (Fig. 2a, Supplemental Tables 1A and B). Only dabigatran had a notable effect on lysis in the TPA-test (Fig. 2b–d; Supplemental Table 1A). The effect of dabigatran was most pronounced with LOT, where the median value of 52 s (IQR 40–63 s) was significantly shorter than that of controls (99 [83–101] seconds; *p* = 0.0086).

Standard laboratory test results are summarized in Supplementary Table 1A. aPTT was significantly increased by UFH and dabigatran versus controls (*p* < 0.0001 for both comparisons). Phenprocoumon was the only anticoagulant with significant impact on INR versus controls (p < 0.0001). Compared with the control group (median 17 [IQR 17–18] seconds), TT was significantly prolonged by dabigatran (144 [114–150] seconds; *p* = 0.0003). Statistically significant prolongation of TT was also observed with UFH (48 s (38–123; *p* = 0.004), although the extent of this prolongation with UFH was much less than that with dabigatran.

## Discussion

This study demonstrates that viscoelastometry is sensitive to a range of anticoagulants, including DOACs. The effects of anticoagulants on the ‘conventional’ EX-test are not specific, while novel parameters (e.g., CT in the RVV-test, CT in the ECA-text, LOT in the TPA-test) could be helpful in differentiating between DOAC classes (direct thrombin inhibitors and FXa-inhibitors).

The direct thrombin inhibitor dabigatran significantly prolonged CT in all five assays compared to controls. Of note, this was the only drug which prolonged CT_ECA-test_, and it was also the only drug which shortened LOT in the TPA-test. FXa inhibitors prolonged CT in all assays except the ECA-test. The magnitude of effect of the FXa inhibitors on CT was less than dabigatran, except in the RVV-test where the median CT was similar in both the dabigatran and FXa-inhibitor groups. Compared with controls, phenprocoumon significantly prolonged CT in the TPA-test and the EX-test, UFH significantly prolonged CT in the IN-test and the RVV-test, and LMWH did not significantly prolong CT in any of the assays.

Correlations observed in this study show that viscoelastometry by ClotPro is suitable for detecting a range of anticoagulants. We observed a strong correlation between CT_ECA-test_ and anti-IIa activity (a surrogate for the concentration of dabigatran). It enabled the calculation of a threshold CT_ECA-test_ value for detecting the presence of dabigatran, and the associated sensitivity and specificity values of 100% show possible capabilities in daily practice. Reliable quantification of dabigatran levels also appears possible, subject to appropriate calibration of CT_ECA-test_. These data are in accordance with our earlier study using the ROTEM delta thromboelastometry device, showing that an ecarin-based test is highly specific and sensitive to direct thrombin inhibitors [19]. Correlation was also observed between CT_RVV-test_ and anti-Xa activity (a surrogate for the concentration of FXa inhibitor or heparin). Further studies are planned to confirm the potential of CT_RVV-test_ for quantifying FXa-inhibitor and LMWH plasma concentrations.

The results of this study suggest that a combination of standard and newly developed viscoelastometric tests like the RVV-test and the ECA-test may provide benefits compared to conventional, standard viscoelastic coagulation monitoring for detection of and differentiation between DOACs [14, 20, 21]. High values for CT_ECA-test_ and CT_RVV-test_ enable confident deduction that a patient is receiving dabigatran (or perhaps a different direct thrombin inhibitor). Prolongation of CT_RVV-test_ but not CT_ECA-test_ is consistent with the patient receiving an FXa inhibitor. However, because CT_RVV-test_ is also prolonged by heparin and (to a lesser extent) by phenprocoumon, it is not clear that the tests performed in this study enable reliable differentiation of FXa inhibitors from other anticoagulant types. Further work is clearly needed in this respect.

In addition to the device used here, there are at least two other devices for point-of-care assessment of DOAC anticoagulation. TEG 6 s is used with cartridges comprising four channels, each with separate reagents to enable four assays to be run simultaneously. In a study performed using an experimental cartridge, detection and classification of DOACs were reported to be achievable with TEG 6 s [15]. The experimental cartridge that was used included one channel with ecarin for detecting dabigatran and another channel with FXa for detecting FXa inhibitors. Assays designed specifically for the detection and classification of DOACs have previously been analyzed using the ROTEM device [19]. Our group recently showed the feasibility of this approach for dabigatran and rivaroxaban [19]. In addition to the sensitivity and specificity of the available tests, a range of other factors (e.g. ease of use, time taken to obtain results, costs, availability) are likely to influence which device is adopted in clinical practice. The ‘active tip’ format of the ClotPro device enables assessments to be performed rapidly with an uncomplicated testing procedure and no requirement for reagent handling by the device user. TEG 6S requires pipetting of blood into the cartridge, whereas ROTEM sigma is a fully automated system. The ClotPro test that is most sensitive to FXa inhibitors (the RVV-test) uses a different approach from that studied by our group with the ROTEM device (TFTEM). In the RVV-test, the snake venom from Daboia Russelli (Russel’s viper venom) is used to trigger coagulation via FX, and this is more specific than the TFTEM assay, which is a diluted EXTEM test. On the other hand, the two ClotPro and ROTEM tests exhibiting high sensitivity to dabigatran (ECA-test and ECATEM) both use ecarin and are therefore similar. In a recently published study by Oberladstätter et al., acutely injured patients on anticoagulant therapy were evaluated using the ClotPro system [22]. The results showed high sensitivity and specificity of the ECA-test for dabigatran and of the RVV-test for FXa inhibitors. The findings of their study are comparable to our results concerning the DOACs in showing the potential application of ClotPro for determining a patient’s anticoagulant treatment. In contrast to the study by Oberladstätter et al., we studied the effects of UFH and LMWH on RVV-test results; strong correlation between CT_RVV-test_ and anti-Xa activity was seen with LMWH and, with UFH, the sensitivity for detection (90%) was as high as that with the IN-test.

Standard laboratory tests in the present study confirmed the presence of clinically relevant plasma concentrations of the anticoagulants. Dabigatran resulted in significant increases in aPTT and TT, while FXa inhibitors had no significant effect on standard laboratory coagulation tests. Phenprocoumon significantly increased INR and, like dabigatran, UFH produced significant increases in aPTT and TT. These results are consistent with previous observations. It has been reported that TT is sensitive to dabigatran but not to FXa inhibitors, while the calibrated anti-Xa test can be used for detection of FXa inhibitors but is not affected by dabigatran [23, 24]. The effects of UFH on aPTT and anti-Xa, and of VKAs such as phenprocoumon on INR, are well known [25, 26].

Limitations of the current study include the modest number of patients, questionable robustness of the sample size calculation (the effect size differs between drugs and tests included in the study), and the lack of blinding. Therefore, further data will be needed to confirm our results. The allowance for variability in dosing and the time between last intake and blood sampling in this study could be considered as further limitations, due to the resulting variability in plasma drug levels. On the other hand, the real-world approach may be considered as a strength of the study because it means the results are applicable to routine clinical practice. Study participants were not selected according to clinical circumstances and the results should be applicable to all patients receiving the drugs included in our study, regardless of the indication for anticoagulant therapy.

## Conclusions

This study has demonstrated that a combination of standard and newly developed viscoelastometric tests on the ClotPro device enable detection of a range of anticoagulants including DOACs. The ECA-test enables detection of dabigatran with 100% specificity and sensitivity. Using RVV-test improves detection of FXa-inhibitors but not differentiation. Quantification of some drugs such as edoxaban, LMWH and dabigatran may be possible; further studies are needed to ascertain whether sufficiently accurate calibration can be achieved. Thus, viscoelastometry may potentially be a useful alternative to standard laboratory methods for the detection and differentiation of anticoagulant drugs.

## Supplementary Information

**Additional file 1 Supplemental Table 1A:** Viscoelastometric and standard laboratory test results from the control group and the anticoagulant groups. Data are presented as median (interquartile range); *p*-values are for comparison with the control group. **Supplemental Table 1B:** Viscoelastometric CT results from the control group and the anticoagulant groups, with pooling of the FXa inhibitors. Data are presented as median (interquartile range); p-values are for comparison with the control group.

## Data Availability

The datasets used and/or analyzed during the current study are available from the corresponding author on reasonable request.

## Abbreviations

A5 / A10: Clot amplitude at 5 and 10 min after CT
Anti-IIa: Anti-activated factor II
Anti-Xa: Anti-activated factor X
aPTT: Activated partial thromboplastin time
AUC: Area under the curve
CFT: Clot formation time
CT: Clotting time
DOAC: Direct oral anticoagulant
ECA-test: Ecarin test
EX-test: Extrinsic test
FIB-test: Functional fibrinogen test
IN-test: Intrinsic test
INR: International normalized ratio
IQR: Interquartile range
LMWH: Low molecular weight heparin
LOT: Lysis onset time
LT: Lysis time
MCF: Maximum clot firmness
ML: Maximum lysis
PCC: Prothrombin complex concentrate
ROC: Receiver operating characteristic
RVV-test: Russel’s viper venom test
SE: Standard error
TPA-test: Tissue plasminogen activator test
TT: Thrombin time
UFH: Unfractionated heparin
VKA: Vitamin K antagonist
