# Supplementary material for: Viscoelastometry for detecting oral anticoagulants
Source: Thromb J. 2021 Mar 16;19:18. doi: 10.1186/s12959-021-00267-w (PMC7962229; doi:10.1186/s12959-021-00267-w)
Supplement: Supplementary file 1 — Additional file 1 Supplemental Table 1A: Viscoelastometric and standard laboratory test results from the control group and the anticoagulant groups. Data are presented as median (interquartile range); p-values are for comparison with the control group. Supplemental Table 1B: Viscoelastometric CT results from the control group and the anticoagulant groups, with pooling of the FXa inhibitors. Data are presented as median (interquartile range); p-values are for comparison with the control group. [file 12959_2021_267_MOESM1_ESM.docx]

**Supplemental Table 1A: Viscoelastometric and standard laboratory test results from the control group and the anticoagulant groups. Data are presented as median (interquartile range); p-values are for comparison with the control group.**

|  | **Controls**  **(n=10)** | **Dabigatran**  **(n=10)** | **Rivaroxaban**  **(n=10)** | **Apixaban**  **(n=10)** | **Edoxaban**  **(n=10)** | **Phenprocoumon**  **(n=10)** | **LMWH**  **(n=10)** | **UFH**  **(n=10)** |
| --- | --- | --- | --- | --- | --- | --- | --- | --- |
| **CT_EX-test_ [s]** | 44  (38-47) | 155  (96-206)  p<0.0001 | 102  (83-110)  p=0.0002 | 52  (47-78)  p>0.9999 | 92  (65-117)  p=0.0016 | 93  (65-106)  p=0.0021 | 50  (45-56)  p>0.9999 | 51  (43-66)  p>0.9999 |
| **A5_EX-test_ [mm]** | 52 (49-54) | 53 (49-58)  p>0.9999 | 54 (51-57)  p>0.9999 | 58 (51-61)  p>0.9999 | 56 (53-62)  p=0.6178 | 58 (54-62)  p=0.2703 | 57  (52-61)  p>0.9999 | 58  (54-64)  p=0.2484 |
| **A10_Ex-test_**  **[mm]** | 59  (58-61) | 60  (58-65)  p>0.9999 | 62  (59-65)  p>0.9999 | 65  (60-69)  p>0.9999 | 64  (61-69)  p=0.5650 | 66  (63-68)  p=0.1976 | 65  (60-68)  p=0.8635 | 66  (61-70)  p=0.2064 |
| **MCF_EX-test_ [mm]** | 62  (62-66) | 64 (62-69)  p>0.9999 | 67  (63-68)  p>0.9999 | 69  (63-72)  p>0.9999 | 67  (64-72)  p>0.9999 | 69  (67-71)  p=0.2397 | 68  (64-72)  p=0.7985 | 69  (67-74)  p=0.1990 |
| **CT_IN-test_ [s]** | 142  (135-148) | 288  (242-336)  p<0.0001 | 200  (183-241)  p=0.0021 | 171  (151-183)  p>0.9999 | 184  (169-220)  p=0.0372 | 162  (149-170)  p>0.9999 | 148  (144-168)  p>0.9999 | 225  (198-276)  p<0.0001 |
| **A5_IN-test_ [mm]** | 49  (47-52) | 49  (47-57)  p>0.9999 | 50  (46-54)  p>0.9999 | 57  (48-59)  p>0.9999 | 52  (48-60)  p>0.9999 | 56  (51-58)  p=0.8956 | 53  (49-58)  p>0.9999 | 52  (47-61)  p>0.9999 |
| **A10_IN-test_ [mm]** | 57  (55-60) | 57  (55-64)  p>0.9999 | 58  (54-62)  p>0.9999 | 64  (57-67)  p>0.9999 | 59  (57-66)  p>0.9999 | 63  (60-65)  p=0.8332 | 61  (55-66)  p>0.9999 | 60  (55-68)  p>0.9999 |
| **MCF_IN-test_ [mm** | 60  (59-65) | 61  (59-68)  p>0.9999 | 63  (59-65)  p>0.9999 | 67  (63-71)  p=0.7435 | 63  (60-71)  p>0.9999 | 67  (64-69)  p=0.4456 | 66  (59-70)  p>0.9999 | 66  (62-72)  p>0.9999 |
| **CT_RVV-test_ [s]** | 63  (58-73) | 200  (148-247)  p=0.0004 | 294  (201-351)  p<0.0001 | 172  (122-232)  p=0.0172 | 203  (177-120)  p=0.0005 | 114  (98-120)  p>0.9999 | 112  (86-123)  p>0.9999 | 289  (214-392)  p<0.0001 |
| **A05_RVV-test_ [mm]** | 52  (48-54) | 49  (48-57)  p>0.9999 | 54  (46-56)  p >0.9999 | 55  (50-59)  p >0.9999 | 53  (51-59)  p >0.9999 | 57  (53-61)  p >0.9999 | 54  (52-60)  p >0.9999 | 49  (44-54)  p >0.9999 |
| **A10_RVV-test_ [mm]** | 60  (57-62) | 58  (56-64)  p>0.9999 | 61  (54-63)  p>0.9999 | 63  (59-67)  p>0.9999 | 61  (60-67)  p>0.9999 | 64  (61-68)  p>0.9999 | 63  (61-67)  p>0.9999 | 58  (53-65)  p>0.9999 |
| **MCF_RVV-test_ [mm]** | 63  (60-67) | 63  (60-67)  p>0.9999 | 66  (59-68)  p>0.9999 | 67  (65-71)  p>0.9999 | 65  (63-72)  p>0.9999 | 69  (66-71)  p=0.9791 | 68  (65-71)  p>0.9999 | 65  (59-70)  p>0.9999 |
| **CT_ECA-test_ [s]** | 73  (70-78) | 307  (246-543)  p=0.0002 | 86  (79-94)  p=0.7412 | 74  (64-79)  p>0.9999 | 78  (71-85)  p>0.9999 | 79  (73-83)  p>0.9999 | 76  (73-80)  p>0.9999 | 71  (62-78)  p>0.9999 |
| **A5_ECA-test_ [mm]** | 54  (50-59) | 51  (46-57)  p>0.9999 | 55  (52-58)  p>0.9999 | 57  (52-61)  p>0.9999 | 57  (52-61)  p>0.9999 | 59  (56-60)  p>0.9999 | 58  (54-58)  p>0.9999 | 58  (54-64)  p>0.9999 |
| **A10_ECA-test_ [mm]** | 61  (59-66) | 60  (58-65)  p>0.9999 | 63  (60-65)  p>0.9999 | 65  (61-69)  p>0.9999 | 64  (60-68)  p>0.9999 | 66  (64-68)  p>0.9999 | 66  (63-68)  p>0.9999 | 66  (61-71)  p>0.9999 |
| **MCF_ECA-test_ [mm]** | 67  (65-72) | 68  (66-72)  p>0.9999 | 68  (66-71)  p>0.9999 | 70  (66-74)  p>0.9999 | 69  (67-73)  p>0.9999 | 72  (70-73)  p>0.9999 | 71  (67-73)  p>0.9999 | 72  (68-75)  p>0.9999 |

| **CT_TPA-test_ [s]** | 39  (35-43) | 147  (94-192)  p<0.0001 | 87  (70-111)  p=0.0007 | 55  (48-79)  p=0.4457 | 92  (76-95)  p=0.0001 | 83  (70-95)  p=0.0007 | 49  (40-56)  p>0.9999 | 54  (44-67)  p>0.9999 |
| --- | --- | --- | --- | --- | --- | --- | --- | --- |
| **A05_TPA-test_ [mm]** | 17  (2-44) | 0  (0-8)  p>0.9999 | 15  (0-38)  p>0.9999 | 43  (20-54)  p>0.9999 | 26  (4-33)  p>0.9999 | 48  (34-56)  p=0.9593 | 40  (19-48)  p>0.9999 | 32  (0-54)  p>0.9999 |
| **A10_TPA-test_ [mm]** | 0  (0-3) | 0  (0-1)  p>0.9999 | 0  (0-0)  p>0.9999 | 0  (0-7)  p>0.9999 | 0  (0-5)  p>0.9999 | 3  (0-30)  p>0.9999 | 0  (0-14)  p>0.9999 | 0  (0-52)  p>0.9999 |
| **MCF_TPA-test_ [mm]** | 38  (34-45) | 25  (21-33)  p>0.9999 | 38  (24-45)  p>0.9999 | 48  (41-55)  p>0.9999 | 43  (30-48)  p>0.9999 | 51  (45-56)  p>0.9999 | 47  (42-54)  p>0.9999 | 48  (36-59)  p>0.9999 |
| **LT_TPA-test_ [s]** | 297  (235-431) | 173  (136-252)  p=0.6161 | 281  (196-374)  p>0.9999 | 422  (295-465)  p>0.9999 | 319  (229-409)  p>0.9999 | 456  (351-610)  p>0.9999 | 379  (298-508)  p>0.9999 | 368  (239-719)  p>0.9999 |
| **LOT_TPA-test_ [s]** | 99  (83-101) | 52  (40-63)  p=0.016 | 86  (69-100)  p>0.9999 | 101  (83-121)  p>0.9999 | 91  (70-102)  p>0.9999 | 101  (87-113)  p>0.9999 | 98  (89-106)  p>0.9999 | 99  (84-116)  p>0.9999 |
| **ML_TPA-test_ [%]** | 96  (95-96) | 93  (92-95)  p>0.9999 | 96  (94-96)  p>0.9999 | 97  (96-97)  p>0.9999 | 96  (95-96)  p>0.9999 | 97  (97-97)  p>0.9999 | 97  (96-97)  p>0.9999 | 97  (96-97)  p>0.9999 |
| **INR** | 1.0  (1.0-1.0) | 1.2  (1.2-1.4)  p=0.0947 | 1.3  (1.2-1.5)  p=0.0541 | 1.1  (0.9-1.2)  p>0.9999 | 1.1  (1.1-1.3)  p>0.9999 | 1.9  (1.8-2.3)  p<0.0001 | 1.0  (0.9-1.0)  p>0.9999 | 1.1  (1.1-1.2)  p>0.9999 |
| **aPTT [s]** | 26  (24-28) | 43  (39-50)  p<0.0001 | 32  (29-35)  p=0.3197 | 27  (25-31)  p>0.9999 | 28  (27-30)  p>0.9999 | 31  (29-25)  p=0.3714 | 29  (24-33)  p>0.9999 | 51  (48-62)  p<0.0001 |
| **Thrombin Time [s]** | 17  (17-18) | 144  (114-150)  p=0.0005 | 17  (16-18)  p>0.9999 | 17  (17-18)  p>0.9999 | 17  (16-18)  p>0.9999 | 18  (17-18)  p>0.9999 | 17  (16-20)  p>0.9999 | 48  (38-123)  p=0.0074 |
| **Anti-IIa activity [ng/ml]** | / | 307  (246-543) | / | / | / | / | / | / |
| **Anti-Xa activity**  **[ng/ml]** | / | / | 188  (89-277) | 87  (56-107) | 88  (61-240) | / | / | / |

A5 / A10, clot amplitude at 5 and 10 minutes, aPTT: activated partial thromboplastin time; CT: clotting time, INR: internationalized normalized ratio; LMWH, low molecular weight heparin; LOT, lysis onset time; LT, lysis time; MCF: maximum clot firmness; ML, maximum lysis, UFH, unfractionated heparin

**Supplemental Table 1B: Viscoelastometric CT results from the control group and the anticoagulant groups, with pooling of the FXa inhibitors. Data are presented as median (interquartile range); p-values are for comparison with the control group.**

| **Test** | **Controls  (n=10)** | **Dabigatran  (n=10)** | **FXa-Inhibitors  (n=30)** | **Phenprocoumon  (n=10)** | **LMWH  (n=10)** | **UFH  (n=10)** |
| --- | --- | --- | --- | --- | --- | --- |
| **CT_EX-test_ [s]** | 44  (38-47) | 155  (96-206)  p<0.0001 | 84  (57-105)  p=0.0003 | 93  (65-106)  p=0.0011 | 50  (45-56)  p>0.9999 | 51  (43-66)  p>0.9999 |
| **CT_IN-test_ [s]** | 142  (135-148) | 288  (242-336)  p<0.0001 | 183  (168-203)  p=0.0026 | 162  (149-170)  p>0.9999 | 148  (144-168)  p>0.9999 | 225  (198-276)  p<0.0001 |
| **CT_RVV-test_ [s]** | 63  (58-73) | 200  (148-247)  p=0.0002 | 207  (159-288)  p<0.0001 | 114  (98-120)  p>0.9999 | 112  (86-123)  p>0.9999 | 289  (214-392)  p<0.0001 |
| **CT_ECA-test_ [s]** | 73  (70-78) | 307  (246-543)  p=0.0001 | 78  (70-86)  p>0.9999 | 79  (73-83)  p>0.9999 | 76  (73-80)  p>0.9999 | 71  (62-78)  p>0.9999 |
| **CT_TPA-test_ [s]** | 39  (35-43) | 147  (94-192)  p<0.0001 | 80  (61-95)  p<0.0001 | 83  (70-95)  p=0.0004 | 49  (40-56)  p>0.9999 | 54  (44-67)  p>0.9999 |

CT: clotting time; LMWH, low molecular weight heparin; UFH, unfractionated heparin
